# Blood concentrates for controlling postoperative complications from third molar surgeries: a scoping review

**DOI:** 10.1590/acb405825

**Published:** 2025-08-08

**Authors:** Vinícius Lima de Almeida, Marcelo Dias Moreira de Assis Costa, Rômulo Dias Jesuino, Lívia Bonjardim Lima, Zuleni Alexandre da Silva, Rafael Rodrigues Lima, Sigmar de Mello Rode, Luiz Renato Paranhos

**Affiliations:** 1 Universidade Federal de Uberlândia - Postgraduate Program in Dentistry - School of Dentistry - Uberlândia (MG), Brazil.; 2 Universidade Federal de Minas Gerais - Department of Oral and Maxillofacial Surgery - School of Dentistry - Belo Horizonte (MG), Brazil.; 3 Universidade Federal de Uberlândia - Department of Oral and Maxillofacial Surgery - School of Dentistry - Uberlândia (MG), Brazil.; 4 Universidade Federal do Pará - Laboratory of Functional and Structural Biology - Institute of Biological Sciences - Belém (PA), Brazil.; 5 Universidade Estadual Paulista - Science and Technology Institute - Department of Dental Materials and Prosthesis - São José dos Campos (SP), Brazil.; 6 Universidade Federal de Uberlândia - Department of Orthodontics - School of Dentistry - Uberlândia (MG), Brazil.

**Keywords:** Biocompatible Materials, Surgery, Oral, Platelet-Rich Fibrin, Postoperative Period, Wound Healing

## Abstract

**Purpose:**

To map the literature on blood concentrates for managing postoperative sequelae after third molar extraction through a scoping review.

**Methods:**

MedLine, Latin American and Caribbean Health Sciences Literature, Scientific Electronic Library Online, EMBASE, Cochrane Library, Scopus, and Web of Science were the search databases. MedRxiv and EASY provided the grey literature. The investigation included observational studies and clinical trials reporting at least one postoperative sequela. After selecting the articles, the extracted data included study author, year, and country, study design, sample size, age distribution, the type of third molar impaction, flap design, the presence of osteotomy and/or odontosection, co-interventions, blood concentrate type, centrifuge model, centrifugation protocol, collection tubes, outcomes, and the main findings for each outcome. After data extraction, an analysis of the geographic distribution of publications was carried out based on the MapChart website.

**Results:**

This review included 63 studies. The leading countries in publications were Turkey, India, and Brazil. Common postoperative sequelae included pain, edema, trismus, alveolar osteitis, and infection. Outcomes varied regarding the effectiveness of concentrates in controlling inflammation, edema, trismus, and alveolar osteitis. Advanced platelet-rich fibrin was the most applied concentrate in recent studies and was associated with reduced edema and trismus.

**Conclusion:**

All concentrates demonstrated some effectiveness in managing sequelae after third molar extraction, but most did not show significant outcomes in controlling inflammatory signs. It emphasizes the need for further randomized clinical trials and systematic reviews to strengthen the evidence on blood concentrates for managing postoperative sequelae.

## Introduction

Tissue repair aims to restore tissue integrity and function after lesions by the coordinated interaction between cells and several chemical mediators. This process starts immediately after the traumatic event and may persist for varied periods^1^. Factors such as third molar extraction may exacerbate the inflammatory response, limiting patients’ daily activities and compromising their postoperative quality of life^2^. This outcome may be attributed to the higher complexity and, sometimes, the need for more invasive surgical techniques to remove these teeth^3^.

Developing strategies for tissue repair optimization to restore tissue physiology and minimize postoperative consequences is an ongoing challenge to improving patients’ quality of life^3^,^4^. Several therapies work for this purpose, each presenting advantages and disadvantages, including anti-inflammatory and analgesic administration^5^, low-level laser therapy^6^, cryotherapy^7^, phytotherapeutics^8^, and blood-derived product application^9^.

The development of platelet-rich blood concentrates originated from collecting platelets and growth factors from the patient’s blood and applying them to surgical sites^9^. The platelet-rich plasma (PRP) was the first-generation blood concentrate^10^-^12^. Later, new blood concentrates emerged, such as platelet-rich fibrin (PRF), whose nomenclature was updated to leukocyte- and PRF (L-PRF)13, advanced platelet-rich fibrin (A-PRF), advanced platelet-rich fibrin plus (A-PRF+), concentrated growth factor (CGF), among others^11^-^14^. These new concentrates stemmed from research that changed collection methods, tube types, the presence or absence of additives in these tubes, centrifugation times, the number of centrifugations, and the g force applied during centrifugations, providing concentrates with different physical, chemical, and biological characteristics^10^.

Blood concentrates are fibrin-based biomaterials containing cells, extracellular matrix proteins, pro- and anti-inflammatory mediators, and growth factors. These biomaterials have favored the management of postoperative sequelae, such as pain, edema, trismus, and alveolar osteitis^15^,^16^. However, assessing this performance has been the target of conflicting analyses in systematic literature reviews, leading to the recommendation of randomized clinical trials with larger samples and more uniform methodologies^17^-^20^.

Therefore, this study proposed a scoping review to investigate the findings of articles related to this topic and identify the main publication niches.

## Methods

### Protocol and registration

This scoping review was conducted following the recommendations of the Joanna Briggs Institute (JBI) manual^21^ and written according to the Preferred Reporting Items for Systematic Reviews and Meta-Analyses extension for Systematic Reviews (PRISMA-ScR)^22^. The review protocol was registered in the Open Science Framework database (https://osf.io/) under registration https://doi.org/10.17605/OSF.IO/ATSU2.

### Eligibility criteria

This review was based on a guiding question created according to the PCC strategy (population, concept, and context) described in the JBI guidelines^21^. The *population* included patients indicated for uni- and bilateral third molar extraction, the concept was clot promotion or blood concentrate application to the socket after extraction, and the *context* was blood concentrate performance against pain, edema, trismus, infection, and alveolar osteitis. That provided the following question: how do blood concentrates perform in controlling inflammatory signs and symptoms after third molar extraction?

This review included observational studies and randomized clinical trials of parallel and split-mouth designs with non-smoker adults without any systemic disease. There was no restriction neither on language nor on year of publication. Studies involving the application of biomaterials with blood concentrates to only one socket were excluded.

### Information sources

The electronic searches occurred up to April 2024 in the MedLine (via PubMed), Latin American and Caribbean Health Sciences Literature (LILACS), Scientific Electronic Library Online (SciELO), EMBASE, Cochrane, Scopus, and Web of Science databases. The MedRxiv and EASY platforms partially captured the grey literature. This search model aimed to minimize the selection bias. The Medical Subject Headings (MeSH), Embase Subject Headings (Emtree), and Health Science Descriptors (DeCS) platforms provided the search descriptors. The Boolean operators “AND” and “OR” combined the keywords, respecting the syntax rules of each database (Table 1).

**Table 1:** Search strategy used in each database.

Databases	Search strategies (April 2024)
Primary databases	
MEDLINE (via PubMed) http://www.ncbi.nlm.nih.gov/pubmed	**#1** “Lower Third Molar” OR “Wisdom Teeth” OR “Wisdom Tooth” OR “Third Molar” **#2** “Pain” OR “Swelling” OR “Trismus” OR “Alveolitis” OR “Alveolar Osteitis” OR “Dry Socket” OR “Socket” OR “Fibrinolytic Alveolitis” OR “Infection” OR “Cellulitis” OR “Abscess” OR “Recovery” OR “Healing” OR “Wound” OR “Outcomes” OR “Efficacy” OR “Morbidities” OR “Comparison” **#3** “PRF” OR “CGF” OR “PRP” OR “Platelet Rich Fibrin” OR “Leukocyte and Platelet Rich Fibrin” OR “Concentrated Growth Factor” OR “Platelet-Rich Plasma” OR “Platelet-Rich” OR “Platelet Rich”
	**#1 AND #2 AND #3**
Embase https://www.embase.com	**#1** “Lower Third Molar” OR “Wisdom Teeth” OR “Wisdom Tooth” OR “Third Molar” **#2** “Pain” OR “Swelling” OR “Trismus” OR “Alveolitis” OR “Alveolar Osteitis” OR “Dry Socket” OR “Socket” OR “Fibrinolytic Alveolitis” OR “Infection” OR “Cellulitis” OR “Abscess” OR “Recovery” OR “Healing” OR “Wound” OR “Outcomes” OR “Efficacy” OR “Morbidities” OR “Comparison” **#3** “PRF” OR “CGF” OR “PRP” OR “Platelet Rich Fibrin” OR “Leukocyte and Platelet Rich Fibrin” OR “Concentrated Growth Factor” OR “Platelet-Rich Plasma” OR “Platelet-Rich” OR “Platelet Rich”
	**#1 AND #2 AND #3**
Scopus http://www.scopus.com/	**#1** “Lower Third Molar” OR “Wisdom Teeth” OR “Wisdom Tooth” OR “Third Molar” **#2** “Pain” OR “Swelling” OR “Trismus” OR “Alveolitis” OR “Alveolar Osteitis” OR “Dry Socket” OR “Socket” OR “Fibrinolytic Alveolitis” OR “Infection” OR “Cellulitis” OR “Abscess” OR “Recovery” OR “Healing” OR “Wound” OR “Outcomes” OR “Efficacy” OR “Morbidities” OR “Comparison” **#3** “PRF” OR “CGF” OR “PRP” OR “Platelet Rich Fibrin” OR “Leukocyte and Platelet Rich Fibrin” OR “Concentrated Growth Factor” OR “Platelet-Rich Plasma” OR “Platelet-Rich” OR “Platelet Rich”
	**#1 AND #2 AND #3**
Cochrane Library https://www.cochranelibrary.com/	**#1** “Lower Third Molar” OR “Wisdom Teeth” OR “Wisdom Tooth” OR “Third Molar” **#2** “Pain” OR “Swelling” OR “Trismus” OR “Alveolitis” OR “Alveolar Osteitis” OR “Dry Socket” OR “Socket” OR “Fibrinolytic Alveolitis” OR “Infection” OR “Cellulitis” OR “Abscess” OR “Recovery” OR “Healing” OR “Wound” OR “Outcomes” OR “Efficacy” OR “Morbidities” OR “Comparison” **#3** “PRF” OR “CGF” OR “PRP” OR “Platelet Rich Fibrin” OR “Leukocyte and Platelet Rich Fibrin” OR “Concentrated Growth Factor” OR “Platelet-Rich Plasma” OR “Platelet-Rich” OR “Platelet Rich”
	**#1 AND #2 AND #3**
Web of Science http://apps.webofknowledge.com/	**#1** “Lower Third Molar” OR “Wisdom Teeth” OR “Wisdom Tooth” OR “Third Molar” **#2** “Pain” OR “Swelling” OR “Trismus” OR “Alveolitis” OR “Alveolar Osteitis” OR “Dry Socket” OR “Socket” OR “Fibrinolytic Alveolitis” OR “Infection” OR “Cellulitis” OR “Abscess” OR “Recovery” OR “Healing” OR “Wound” OR “Outcomes” OR “Efficacy” OR “Morbidities” OR “Comparison” **#3** “PRF” OR “CGF” OR “PRP” OR “Platelet Rich Fibrin” OR “Leukocyte and Platelet Rich Fibrin” OR “Concentrated Growth Factor” OR “Platelet-Rich Plasma” OR “Platelet-Rich” OR “Platelet Rich”
	**#1 AND #2 AND #3**
Latin American and Caribbean Health Sciences Literature (LILACS) http://pesquisa.bvsalud.org/	(("Lower Third Molar" OR "Wisdom Teeth" OR "Wisdom Tooth" OR "Third Molar") AND (“Pain” OR “Swelling” OR “Trismus” OR “Alveolitis” OR “Alveolar Osteitis” OR “Dry Socket” OR “Socket” OR “Fibrinolytic Alveolitis” OR “Infection” OR “Cellulitis” OR “Abscess” OR “Recovery” OR “Healing” OR “Wound” OR “Outcomes” OR “Efficacy” OR “Morbidities” OR “Comparison”) AND ("PRF" OR "CGF" OR "PRP" OR "Platelet Rich Fibrin" OR "Leukocyte and Platelet Rich Fibrin" OR "Concentrated Growth Factor" OR "Platelet-Rich Plasma" OR "Platelet-Rich" OR "Platelet Rich"))
Scientific Electronic Library Online (SciELO) https://scielo.org/	(("Lower Third Molar" OR "Wisdom Teeth" OR "Wisdom Tooth" OR "Third Molar") AND (“Pain” OR “Swelling” OR “Trismus” OR “Alveolitis” OR “Alveolar Osteitis” OR “Dry Socket” OR “Socket” OR “Fibrinolytic Alveolitis” OR “Infection” OR “Cellulitis” OR “Abscess” OR “Recovery” OR “Healing” OR “Wound” OR “Outcomes” OR “Efficacy” OR “Morbidities” OR “Comparison”) AND ("PRF" OR "CGF" OR "PRP" OR "Platelet Rich Fibrin" OR "Leukocyte and Platelet Rich Fibrin" OR "Concentrated Growth Factor" OR "Platelet-Rich Plasma" OR "Platelet-Rich" OR "Platelet Rich"))
**Grey literature**	
MedRxiv https://www.medrxiv.org	“Third Molar” AND “Platelet Rich Fibrin”
EASY https://easy.dans.knaw.nl/	“Third Molar” AND “Platelet Rich Fibrin”

The results from the primary databases were exported to EndNote Web software (Clarivate Analytics, Philadelphia, United States of America). The program automatically excluded duplicates, and the remaining ones were manually removed. The remaining findings were exported to Rayyan Qatar Computing Research Institute (QCRI, Doha, Qatar) for selecting and evaluating titles and abstracts^23^. The grey literature was manually, simultaneously, thoroughly analyzed, and exported to Microsoft Word 2010 (Microsoft Ltd., Washington, United States of America).

Two reviewers (VLA and MDMAC) discussed the inclusion and exclusion criteria before selecting the studies, then performed the calibration exercise by assessing 20% of articles to determine evaluator agreement. Selection started after reaching an adequate level of agreement (Kappa ≥ 0.81).

In the first review step, two examiners (VLA and MDMAC) evaluated the titles independently, according to the eligibility criteria. The titles unrelated to the review proposal were discarded. Next, the reviewers assessed the abstracts independently, according to the eligibility criteria. Finally, the eligible studies were fully read, following the same process as the previous steps. A third examiner (LRP) helped assess and solve reviewer disagreements in all stages.

### Data charting process

After selecting the articles, the reviewers performed a calibration exercise to ensure consistency, coherence, and agreement in data extraction. They jointly collected data from three eligible studies in this exercise. Then, two evaluators (VLA and MDMAC) independently extracted the data from the eligible articles. A third reviewer (LRP) analyzed conflicts, and the researchers discussed them.

### Data items

The extracted data included study author, year, and country, study design, sample size, age distribution, the type of third molar impaction, flap design, the presence of osteotomy and/or odontosection, co-interventions, blood concentrate type, centrifuge model, centrifugation protocol, collection tubes, outcomes, and the main findings for each outcome. After data extraction, an analysis of the geographic distribution of publications was carried out based on the MapChart website.

In cases of data unavailability, the authors of the included studies were contacted via e-mail. Regarding missing or incomplete data and the absence of further clarification from the authors, a “no information” notation was applied.

## Results

### Selection of sources of evidence

**Figure 1:**
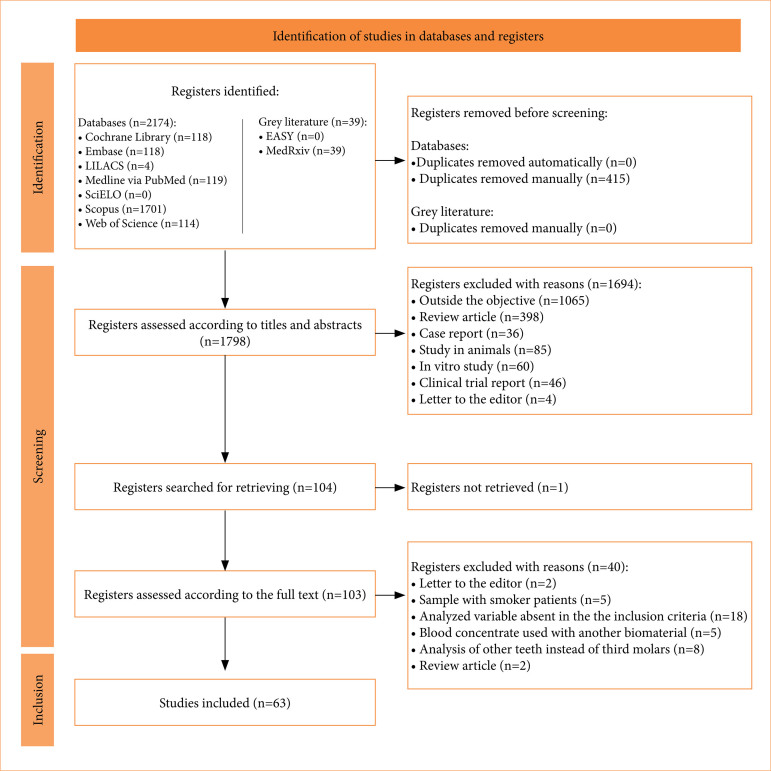
Flow diagram of literature search and selection criteria adapted from Preferred Reporting Items for Systematic Reviews and Meta-Analyses extension for Systematic Reviews (PRISMA). LILACS: Latin American and Caribbean Health Sciences Literature; SciELO: Scientific Electronic Library Online.

Source: Elaborated by the authors.

The initial phase of study selection yielded 2,213 results across nine electronic databases, including the grey literature. After duplicate removals, 1,798 results remained for title and abstract screening. These studies underwent evaluation, with 104 selected for a full-text assessment, but one study could not be retrieved. Consequently, 103 articles underwent a full-text review and were subjected to the eligibility criteria. Subsequently, articles unrelated to the topic or failing to meet the inclusion criteria were excluded, leaving 63 studies. Figure 1 illustrates the search, identification, inclusion, and exclusion of eligible studies.

### Synthesis of results

Table 2 presents the general characteristics of the eligible studies. Concerning study type, one was a pilot study^24^, one was observational retrospective^31^, one was observational, prospective, and non-randomized^44^, two were quasi-experimental^25^,^28^, and the remaining 58 were randomized clinical trials with split-mouth or parallel designs. Approximately half the studies determined third molar position classification according to Winter and/or Pell and Gregory, and the others only stated whether they were impacted or did not provide precise information regarding their positioning in maxillary bones. All articles were in English, and only two examined blood concentrate performance after upper third molar extraction^44^,^82^, while the others solely included lower third molars.

**Table 2:** General characteristics of eligible studies.

Authors, year of publication, country	Study design	Sample size	Age range (mean-SD)	Type of third molar	Flap design	Bone remo- val	Co- interventions	Blood concentrate	Centrifuge	Blood concentrate protocol	Tubes	Outcomes
Daugela et al., 2018^16^ Lithuania	Randomized clinical trial (Split-mouth	14 ♂ 20 ♀	18-60 (22.76 ± 2.02)	Impacted mandibular third molars	Triangular flap	Yes	NSAID	L-PRF	n. r.	2,800 for 12 min	9-mL glass-coated plastic tubes (plastic) [Intra-Spin, Intra-lock international]	Pain and swelling
Gawande and Halli, 2009^24^ India	Pilot study	20	18-30 **	Impacted mandibular third molars	n. r.	n. r.	n. r.	PRP	Digital centrifugation machine (REMI Motors Ltd.)	1,200 rpm for 10 min and 2,000 rpm for 10 min	5-mL Vacutainer tubes with citrate phosphate dextrose adenine (no material) [Becton & Dickinson]	Pain and swelling
Vivek and Sripathi Rao, 2009^25^ India	Quasi-experimental clinical trial (Split-mouth)	5 ♂ 5 ♀	18-45 (27) **	Mandibular third molars (unclassified)	n. r.	n. r.	n. r.	PRP	n. r.	n. r.	10-mL tubes (no material) [no brand]	Pain
Arenaz-Búa et al., 2010^26^ Spain	Randomized clinical trial (Split-mouth)	34 *	**	Mandibular third molars (unclassified)	n. r.	n. r.	ATB, NSAID, and ANA	PRP	n. r.	n. r.	n. r.	Pain, edema, trismus, and infection
Mozzati et al., 2010^27^ Italy	Randomized clinical trial (Split-mouth)	16 *	18-35 **	Impacted mandibular third molars (unclassified)	Envelope flap	Yes	ATB and NSAID	PRGF	n. r.	1,800 rpm for 8 min	5-mL tubes with 3.8% trisodium citrate (no material) [no brand]	Pain and swelling
Rutkowski et al., 2010^28^ United States of America	Quasi-experimental clinical trial (Split-mouth)	6 *	18-40 **	Mandibular third molars (unclassified)	Envelope flap	Yes	ATB, NSAID, and ANA	PRP	n. r.	10 min at 1,150 g	4.5-mL Vacutainer tubes with trisodium citrate (no material) [Becton & Dickinson]	Pain and swelling
Ogundipe et al., 2011^29^ Nigeria	Randomized clinical trial (Parallel)	25 ♂ 35 ♀	19-35 (24.7 ± 3.6)	31 mesioangular ones, 14 distoangular ones, 9 verticals, and 6 horizontals	Triangular flap	Yes	n. r.	PRP	PowerSpin Fx centrifuge (Single)	1,200 rpm for 10 min and 1,000 rpm for 10 min	10-mL tubes with citrate phosphate dextrose (no material) [no brand]	Pain, swelling, and trismus
Singh et al., 2012^30^ India	Randomized clinical trial (Split-mouth)	10 ♂ 10 ♀	18-50 **	Mandibular third molars (unclassified)	n. r.	n. r.	n. r.	L-PRF	n. r.	3,000 rpm for 10 min	10-mL tubes (no material) [no brand]	Pain
Hoaglin and Lines, 2013^31^ United States of America	Retrospective study (Parallel)	187 *	14-40 **	Mandibular third molars (unclassified)	n. r.	n. r.	ATB, NSAID, and ANA	L-PRF	n. r.	2,700 rpm for 12 min	8.5-10-mL Vacutainer tubes without citrate (no material) [Becton & Dickinson]	Alveolar osteitis
Eshghpour et al., 2014^32^ Iran	Randomized clinical trial (Split-mouth)	33 ♂ 45 ♀	18-35 (25.09 ± 4.25)	Bilaterally impacted mandibular third molars with similar impaction (Peterson›s classification)	Envelope flap	Yes	ATB and ANA	L-PRF	Labofuge Centrifuge 400R (Heraeus, Hanau, Germany)	3,000 rpm for 10 min	10-mL tubes (no material) [no brand]	Alveolar osteitis
Gawai and Sobhana, 2015^33^ India	Randomized clinical trial (Split-mouth)	3 ♂ 2 ♀	19-32 (22.8 + 5.2)	Impacted mandibular third molars (unclassified)	Envelope flap	Yes	ATB and ANA	PRP	Laboratory centrifuge R-8C REMI	2,400 rpm for 10 min and 3,600 for 10 min	10-mL tubes with citrate phosphate dextrose (no material) [no brand]	Pain and swelling
Kumar et al., 2015^34^ India	Randomized clinical trial (Parallel)	31 *	19-35 **	19 mesioangular ones and 12 horizontals	Envelope flap	Yes	ATB, SAID, NSAID, and ANA	L-PRF	n. r.	3,000 rpm for 10 min	5-mL tubes (no material) [no brand]	Pain, swelling, and trismus
Ozgul et al., 2015^35^ Turkey	Randomized clinical trial (Split-mouth)	23 ♂ 33 ♀	18-28 **	20 horizontals, 15 mesioangular ones, and 21 verticals	Envelope and triangular flaps	Yes	ATB and ANA	L-PRF	n. r.	3,000 rpm for 10 min	10-mL glass-coated plastic tubes (plastic) [no brand]	Pain and swelling
Uyanik et al., 2015^36^ Cyprus	Randomized clinical trial (Split-mouth)	10 ♂ 10 ♀	19-31 **	Bilaterally impacted mandibular third molars in vertical position (Pell and Gregory’s IC classification)	Triangular flap	Yes	ATB and ANA	L-PRF	Elektro-mag M415P (Istanbul, Turkey)	3,000 rpm for 10 min (400 g)	10-mL glass-coated plastic tubes (plastic) [no brand]	Pain, swelling, and trismus
Bilginaylar and Uyanik, 2016^37^ Cyprus	Randomized clinical trial (Split-mouth and Parallel)	22 ♂ 37 ♀	18-31 **	Bilaterally impacted mandibular third molars in vertical position (Pell and Gregory’s IC classification)	Triangular flap	Yes	ATB and ANA	L-PRF	Elektro-mag M415P (Istanbul, Turkey)	3,000 rpm for 10 min (400 g)	10-mL glass-coated plastic tubes (plastic) [no brand]	Pain, swelling, and trismus
Dutta et al., 2016^38^ India	Randomized clinical trial (Parallel)	30 *	**	Mandibular third molars (unclassified)	Envelope and triangular flaps	Yes	ATB and ANA	PRP and L-PRF	n. r.	PRP: 2,000 rpm for 15 min and 3,000 rpm for 10 min PRF: 2,000 rpm for 15 min	PRP: first centrifugation-4-mL tubes with citrate phosphate dextrose adenine solution (no material) [no brand]. Second centrifugation-test tube (no material) [no brand]	Pain, swelling, and alveolar osteitis
Kumar et al., 2016^39^ India	Randomized clinical trial (Split-mouth)	8 *	18-40 **	Mandibular third molars (unclassified)	Triangular flap	Yes	ATB and ANA	L-PRF	n. r.	3,000 rpm for 10 min	PRF: test tube (no material) [no brand]	Pain and infection
Al-Hamed et al., 2017^40^ Egypt	Randomized clinical trial (Parallel)	13 ♂ 37 ♀	18-48 **	Vertical, mesioangular, horizontal, and distoangular ones	Envelope flap	Yes	ATB, NSAID, and AMW	L-PRF	80-1 electric centrifuge	3,000 rpm for 10 min	n. r.	Pain
Asutay et al., 2017^41^ Turkey	Randomized clinical trial (Split-mouth)	6 ♂ 24 ♀	18-29 (20.32)	Bilaterally impacted mandibular third molars in mesioangular position	Triangular flap	Yes	ATB and ANA	L-PRF	n. r.	2,700 for 12 min	5-mL tubes without anticoagulant (no material) [no brand]	Pain, swelling, and trismus
Gandevivala et al., 2017^42^ India	Randomized clinical trial (Split-mouth)	25 *	16-60 **	n. r.	Triangular flap	Yes	n. r.	PRP	n. r.	1,300 rpm for 10 min and 1,200 rpm for 10 min	10-mL tubes without anticoagulant (no material) [no brand]	Pain and swelling
Gülşen and Şentürk, 2017^43^ Turkey	Randomized clinical trial (Split-mouth)	21 ♂ 9 ♀	17-27 (20.03)	Vertical (Pell and Gregory's IB classification)	Triangular flap	Yes	ATB, NSAID and AMW	L-PRF	NUVE NF 200	3,000 rpm for 10 min	First centrifugation: 10-mL tubes with citrate phosphate dextrose adenine solution (no material) [no brand]. Second centrifugation: no information	Pain and swelling
Rastogi et al., 2018^44^ India	Observational, prospective, and non-randomized	21 ♂ 79 ♀	18-40 **	Maxillary and mandibular third molars (unclassified)	n. r.	n. r.	n. r.	L-PRF	R-8C BL, REMI (Labs, India)	3,000 rpm for 12 min	10-mL glass-coated plastic tubes (Poly Medicure Ltd., New Delhi, India)	Pain
Afat et al., 2018^45^ Turkey	Randomized clinical trial (Parallel)	15 ♂ 25 ♀	18-30 **	(Pell and Gregory's IIB classification)	n. r.	Yes	n. r.	L-PRF	Elektro-mag M615P (Turkey)	3,000 rpm for 12 min	10-mL tubes (glass) [no brand]	Pain, swelling, and trismus
Bhujbal et al., 2018^46^ Iran	Randomized clinical trial (Split-mouth)	5 ♂ 15 ♀	25.2 ± 7.19	Impacted mandibular third molars	Triangular flap	Yes	n. r.	PRP	Elektro-mag M615P (Turkey)	1,200 rpm for 10 min and 2,000 rpm for 10 min	Coated plastic tubes (plastic) [Becton & Dickinson]	Pain and swelling
Dar et al., 2018^47^ India	Randomized clinical trial (Split-mouth)	13 ♂ 17 ♀	18-50 (23.6 ± 4.3)	Mesioangular	Triangular flap	Yes	ATB and NSAID	L-PRF	n. r.	3,000 rpm for 12 min	10-mL tubes without anticoagulant (no material) [no brand]	Pain and swelling
Eshghpour et al., 2018^48^ Iran	Randomized clinical trial (Split-mouth)	49 ♂ 69 ♀	18-35 (23.94 ± 3.77)	Impacted mandibular third molars	Envelope flap	Yes	ATB and ANA	L-PRF	Labofuge Centrifuge 400R (Heraeus, Hanau, Germany)	3,000 rpm for 10 min	10-mL tubes (no material) [no brand]	Alveolar osteitis
Jeyaraj and Chakranarayan, 2018^49^ India	Randomized clinical trial (Parallel)	60 n. r.	**	n. r.	n. r.	Yes	ATB, NSAID, and ANA	L-PRF	Hettich Zentrifugen Centrifuge EBA 20 (Andreas Hettich GmbH& Co., KG, Germany)	2,700 rpm for 12 min	12-mL tubes (borosilicate glass tube) [no brand]	Pain, swelling, and trismus
Afat et al., 2019^50^ Turkey	Randomized clinical trial (Parallel)	15 ♂ 25 ♀	18-30 **	(Pell and Gregory's IIB classification)	n. r.	n. r.	ATB, ANA, and AMW	L-PRF	Elektro-mag M615P (Istanbul, Turkey)	3,000 rpm (400 g) for 10 min	10-mL glass-coated plastic tubes (plastic) [Becton & Dickinson]	Alveolar osteitis and infection
Caymaz and Uyanik, 2019^51^ Turkey	Randomized clinical trial (Split-mouth)	12 ♂ 15 ♀	18-26 **	(Pell and Gregory's IC classification)	Triangular flap	Yes	ATB, NSAID, and AMW	L-PRF and A-PRF	n. r.	L-PRF: 3,000 rpm for 10 min A-PRF: 1,500 rpm for 14 min	10-mL tubes without anticoagulant (no material) [no brand]	Pain, swelling, and trismus
Kapse et al., 2019^52^ India	Randomized clinical trial (Split-mouth)	13 ♂ 17 ♀	18-40 (25.47 ± 0.9)	Impacted mandibular third molars	n. r.	Yes	n. r.	L-PRF	Elektro-mag	2,700 rpm for 12 min	9-mL tubes without anticoagulant (no material) [Intra Lock]	Pain and swelling
Ritto et al., 2019^53^ Brazil	Randomized clinical trial (Split-mouth)	10 ♂ 7 ♀	16-29 (21.8)	n. r.	Envelope flap	Yes	NSAID, SAID, and ANA	L-PRF	Elektro-mag M 415 P (Turkey)	2,700 rpm for 12 min	10-mL tubes without anticoagulant (no material) [no brand]	Pain
Zahid and Nadershah, 2019^54^ Saudi Arabia	Randomized clinical trial (Split-mouth)	10 ♀	** (24)	Mesioangular and vertical mandibular third molars	Envelope flap	n. r.	ATB, NSAID, and AMW	A-PRF	R-4C DX REMI	1,300 rpm for 13 min	10-mL tubes (glass) [no brand]	Pain and swelling
Aftab et al., 2020^55^ India	Randomized clinical trial (Parallel)	38 ♂ 62 ♀	18-42 (26.12 ± 5.92)	(Pell and Gregory's B classification)	Envelope flap	Yes	n. r.	PRP	A-PRF 12	1,200 rpm for 10 min and 1,000 rpm for 10 min	Coated plastic tubes (plastic) [Becton & Dickinson]	Pain, swelling, and trismus
Bhujbal et al., 2020^56^ India	Randomized clinical trial (Split-mouth)	8 ♂ 12 ♀	22 (26 ± 6.9) **	n. r.	Triangular flap	Yes	ATB and NSAID	PRP and L-PRF	DUO centrifuge (Process for PRF, Nice, France)	PRP: 1,200 rpm for 10 min and 2,000 rpm for 10 min PRF: 3,000 rpm for 10 min	A-PRF: 10-mL tubes without anticoagulant (glass) [A-PRF+ by Choukroun]	Pain and swelling
Özveri Koyuncu et al., 2020^57^ Turkey	Randomized clinical trial (Split-mouth)	26 ♂ 44 ♀	18-35 (25.86)	Vertical mandibular third molars (Pell and Gregory's IB classification)	Envelope flap	Yes	ATB and NSAID	CGF	Medifuge (France)	2,700 rpm for 2 min, 2,400 rpm for 4 min, and 2,700 rpm for 4 min	9-mL Vacutainer glass-coated tubes (glass) [no brand]	Alveolar osteitis
Özveri Koyuncu et al., 2020^58^ Turkey	Randomized clinical trial (Split-mouth)	21 ♂ 39 ♀	18-35 (25.82)	Vertical mandibular third molars (Pell and Gregory's IB classification)	Envelope flap	Yes	ATB and NSAID	CGF	Medifuge (France)	2,700 rpm for 2 min, 2,400 rpm for 4 min, and 2,700 rpm for 4 min	9-mL Vacutainer glass-coated tubes (glass) [no brand]	Pain, swelling, and trismus
Kumar et al., 2020^59^ India	Randomized clinical trial (Split-mouth)	68 ♂ 32 ♀	20-50 **	(Pell and Gregory's IA or IB classification)	n. r.	n. r.	ATB and NSAID	PRP	n. r.	2,000 rpm for 15 min and 3,000 rpm for 10 min	10-mL tubes without anticoagulant (no material) [no brand]	Pain, swelling, and trismus
Miyamoto et al., 2020^60^ Japan	Randomized clinical trial (Split-mouth)	14 ♂ 18 ♀	20-51	17 IA, 5 IB, 15 2A, 23 IIB, and 4 IIC (Pell and Gregory's classification)	n. r.	Yes	ATB and NSAID	L-PRF	Medifuge (France)	400 g for 10 min	10-mL tubes without anticoagulant (glass) [A-PRF+ by Choukroun]	Pain and swelling
Sybil et al., 2020^61^ India	Randomized clinical trial (Split-mouth)	14 ♂ 11 ♀	18-55 (32.3)	30 mesioangular and 50 horizontal mandibular third molars	n. r.	n. r.	n. r.	L-PRF	n. r.	3,000 rpm for 10 min	10-mL tubes with citrate (no material) [no brand]	Pain and swelling
Torul et al., 2020^62^ Turkey	Randomized clinical trial (Parallel)	23 ♂ 52 ♀ SG 1: 25 SG 2: 25 CGF: 25	18-37 (22.31 ± 4.65)	Impacted mandibular third molar. Radiographic degree of the impacted tooth was mesioangular according to Winter’s classification and II, or III/B according to Pell and Gregory’s classification)	n. r.	Yes	n. r.	SG 1: A-PRF SG 2: CGF	n. r.	1.300 rpm for 14 min (A-PRF), 2,700 rpm for 2 min, 2,400 rpm for 4 min, 2,700 rpm for 4 min, and 3,000 rpm for 3 min (CGF)	PRP: first centrifugation-6-mL tubes with citrate phosphate dextrose adenine (no material) [no brand]	Pain, swelling, and trismus
Gupta and Agarwal, 2021^63^ India	Randomized clinical trial (Split-mouth)	18 ♂ 12 ♀	18-35 **	n. r.	Triangular flap	Yes	n. r.	A-PRF	n. r.	1,500 rpm for 14 min	Test tubes without anticoagulant (no material) [no brand]	Pain, swelling, and trismus
Hanif and Sheikh, 2021^64^ Pakistan	Randomized clinical trial (Parallel)	52 ♂ 78 ♀	19-43 (27.95 ± 6.05)	n. r.	n. r.	n. r.	ANA	PRP	A-PRF 12	1,200 rpm for 10 min and 2,000 rpm for 10 min	Vacuum tube coated with anticoagulant (no material) [no brand]	Pain and trismus
Nourwali, 2021^65^ Saudi Arabia	Randomized clinical trial (Parallel)	20 *	18-40 **	n. r.	Envelope flap	Yes	ATB, NSAID, and SMW	L-PRF	Power Spin UNICO	3,000 rpm for 10 min	10-mL tubes without anticoagulant (no material) [no brand]	Pain and swelling,
da Silva et al., 2021^66^ Brazil	Randomized clinical trial (Split-mouth)	6 ♂ 14 ♀	18-29 (23 ± 3.28)	Mandibular third molar (Pell and Gregory's IA classification)	Envelope flap	n. r.	NSAID, ANA, and AMW	L-PRF	IntraSpin, Biohorizons (Birmingham, Alabama, AL, United States of America)	2,700 rpm for 12 min	10-mL tubes without anticoagulant (no material) [Becton & Dickinson]	Pain
Nowak et al., 2021^67^ Poland	Randomized clinical trial (Parallel)	38 ♂ 22 ♀	18-40 **	Mesioangular mandibular third molars (Pell and Gregory's IIB classification)	Envelope flap	Yes	ATB and NSAID	L-PRF	PRF Duo Quattro	1,500 rpm for 14 min	10-mL tubes (no material) [4 A-x BY CHOUKROUN]	Pain, swelling, and trismus
Starzyńska et al., 2021^68^ Poland	Randomized clinical trial (Parallel)	35 ♂ 65 ♀	18-40 **	58 vertical and 42 horizontal mandibular third molars	Triangular flap	Yes	ATB and ANA	A-PRF	All Centrifuge, Scilogex, LLC (Rocky Hill, CT, United States of America)	1,500 rpm for 14 min	10-mL glass-coated plastic tubes (plastic) [no brand]	Pain, swelling, trismus, and alveolar osteitis
Trybek et al., 2021^69^ Poland	Randomized clinical trial (Parallel)	28 ♂ 62 ♀	18-37 **	n. r.	Triangular flap	Yes	NSAID and AMW	L-PRF	EBA-200 (Hettich, Germany)	2,700 rpm for 12 min	10-mL tubes (no material) [no brand]	Pain, swelling, and trismus
Elayah et al., 2022^70^ China	Randomized clinical trial (Split-mouth)	19 ♂ 18 ♀	18-38 (25)	Vertical and horizontal impacted mandibular third molars	Triangular flap	Yes	ATB, ANA, and AMW	CGF	CGF centrifuge equipment (Trausim, DL4015, Dental Regenerative Centrifuge, China)	2,300 rpm for 13 min	10-mL tubes without anticoagulant (no material) [no brand]	Pain and swelling
Fang et al., 2022^71^ China	Randomized clinical trial (Parallel)	66 ♂ 52 ♀	18-50 (30)	Mesioangular, horizontal, vertical, and Pell and Gregory's II B mandibular third molars	Envelope flap	Yes	n. r.	CGF	Medifuge centrifugal accelerator (Salfident, Bologna, Italy)	2,700 rpm for 4 min, 2,400 rpm for 4 min, and 2,700 rpm for 3 min	10-mL tubes without anticoagulant (no material) [no brand]	Pain, swelling, trismus, and alveolar osteitis
Konuk and Senturk, 2022^72^ Turkey	Randomized clinical trial (Split-mouth)	8 ♂ 22 ♀	18-28 (20.36 ± 2.85)	Vertical mandibular third molars (Pell and Gregory’s IA or IB classification)	Triangular flap	Yes	ATB, NSAID, and AMW	L-PRF	Hettich EBA 20	3,000 rpm for 10 min	2-9-mL tubes without anticoagulant (no material) [Vacuette]	Swelling
Osagie et al., 2022^73^ Nigeria	Randomized clinical trial (Parallel)	17 ♂ 33 ♀	18-55 (26.5 ± 7.8)	(Pell and Gregory's IIB classification)	Triangular flap	Yes	ATB and NSAID	PRP and L-PRF	n. r.	PRP: 1,338 rpm (100 g) and 2,675 rpm (400 g) PRF: 2,675 rpm (400 g)	3.5-mL glass coated test tube with 3.2% sodium citrate (no material) [no brand]	Pain, swelling, and trismus
O’Sullivan et al., 2022^74^ Ireland	Randomized clinical trial (Parallel)	17 ♂ 57 ♀	19-39 (28.1 ± 5.8)	Impacted mandibular third molars	Envelope flap	Yes	NSAID, ANA, and AMW	PRGF	n. r.	500 g for 8 min	9-mL tubes with 3.8% trisodium citrate (no material) [no brand]	Pain, trismus, and alveolar osteitis
Rengarajoo et al., 2022^75^ Malaysia	Randomized clinical trial (Split-mouth)	3 ♂ 12 ♀	21 - 35 (26 ± 4.9)	Impacted mandibular third molars	n. r.	n. r.	n. r.	LPRP	n. r.	n. r.	10-mL tubes (no material) [Becton & Dickinson]	Pain, swelling, and trismus
Riaz et al., 2022^76^ India	Randomized clinical trial (Split-mouth)	3 ♂ 7 ♀	18-35 (26.5)	Impacted mandibular third molars	Triangular flap	Yes	ATB, NSAID, and ANA	L-PRF and A-PRF	REMI C-852, Remielektrotechnik Ltd. (Vasai, India)	L-PRF: 3,000 rpm for 10 min. A-PRF: 1,500 rpm for 14 min	5-mL tubes without anticoagulant (no material) [no brand]	Pain, swelling, and trismus
Sargaiyan et al., 2022^77^ India	Randomized clinical trial (Split-mouth)	7 ♂ 8 ♀	18-35 (25.16)	21 mesioangular ones, 5 distoangular ones, and 4 vertical mandibular third molars	n. r.	n. r.	ATB, NSAID, and AMW	PRP	n. r.	n. r.	n. r.	Pain, swelling, and alveolar osteitis
Shruthi et al., 2022^78^ India	Randomized clinical trial (Parallel)	44 *	18-40 **	n. r.	Triangular flap	Yes	n. r.	L-PRF	n. r.	n. r.	n. r.	Pain, swelling, and trismus
Asif et al., 2023^79^ Saudi Arabia	Randomized clinical trial (Parallel)	90 ♂ 90 ♀	18-65 (41.35 ± 9.87)	n. r.	Envelope flap	Yes	ATB and NSAID	L-PRF	n. r.	3,500 rpm for 10 min (1.096 g)	10-mL tubes (glass) [no brand]	Pain and alveolar osteitis
Javid et al., 2023^80^ Brazil	Randomized clinical trial (Split-mouth)	3 ♂ 7 ♀	> 18 22.1 ± 3.14	Pell and Gregory’s IA or IB mandibular third molars	Envelope flap	n. r.	ATB, ANA, and AMW	L-PRF and Alb-PRF	IntraSpin, Biohorizons (Birmingham, AL, United States of America)	L-PRF: 2,700 rpm for 12 min (708 g)	9-mL tubes without anticoagulant (no material) [Becton & Dickinson]	Pain, swelling, and trismus
Karaca et al., 2023^81^ Turkey	Randomized clinical trial (Split-mouth)	17 ♂ 31 ♀	18-41 (24.5 ± 4.5)	Vertical or mesioangular mandibular third molars (Pell and Gregory’s IIB or IIC classification)	Triangular flap	Yes	ATB, ANA, and AMW	L-PRF	Intra-Lock International Inc. (Boca Raton, FL, United States of America)	2,700 rpm for 12 min	10-mL tubes without anticoagulant (no material) [no brand]	Pain, swelling, and trismus
Pereira et al., 2023^82^ Brazil	Randomized clinical trial (Split-mouth)	16 *	> 18 **	Winter: 26 vertical, 2 mesioangular, and 4 distoangular maxillary third molars. Pell and Gregory: 14 A, 10 B, and 8 C	Envelope flap	Yes	ATB, NSAID, ANA and AMW	A-PRF+	SpinPlus Titan, SpinLab (Ribeirão Preto, SP, Brazil)	1,300 for 8 min (200 g)	9-mL silica-coated tubes (plastic) [no brand]	Pain and swelling
Rodrigues et al., 2023^83^ Brazil	Randomized clinical trial (Split-mouth)	7 ♂ 15 ♀	18-28 (22.41 ± 2.74)	16 IB, 12 IC, 8 IIA, and 2 IIB according to Pell and Gregory’s classification, and 6 horizontals	Envelope flap	Yes	ATB, ANA, and AMW	L-PRF	Daiki DT4000	2,700 for 12 min (400 g)	10-mL tubes without anticoagulant (no material) [no brand]	Pain and swelling
Kalyani et al., 2023^84^ India	Randomized clinical trial (Split-mouth)	18-45 *	18-45	Impacted mandibular third molars	Triangular flap	Yes	n. r.	L-PRF	n. r.	2,800 rpm for 12 min	n. r.	Pain and swelling
Akpinar and Ayrancı, 2024^85^ Turkey	Randomized clinical trial (Split-mouth)	9 ♂ 26 ♀	18-25 **	Mesioangular (Winter) and Pell and Gregory's IIB or IC mandibular third molars	Envelope flap	Yes	ATB, ANA, AMW	i-PRF	Intraspin Centrifuge Intra-Lock International Inc. (Boca Raton, FL, United States of America)	700 rpm for 3 min	9-mL tubes without anticoagulant (no material) [no brand]	Pain, swelling, and trismus

♂ male;

♀ female;

* it was impossible to measure the sample by sex;

** it was impossible to measure the range or standard deviation;

n. r.: not reported; ATB: antibiotic; NSAID: non-steroidal anti-inflammatory drug; SAID: steroidal anti-inflammatory drug; ANA: analgesic; AMW: antiseptic mouthwash; SMW: saline mouthwash; SD: standard deviation; g: relative centrifugal force; PRP: platelet-rich plasma; L-PRF: leukocyte- and platelet-rich fibrin; A-PRF: advanced-platelet-rich fibrin; A-PRF+: advanced-platelet-rich fibrin plus; CGF: concentrated growth factor; Alb-PRF: albumin with platelet-rich fibrin; i-PRF: injectable platelet-rich fibrin; PRGF: platelet-rich growth factor; LPRP: lyophilized platelet-rich plasma.

**Figure 2:**
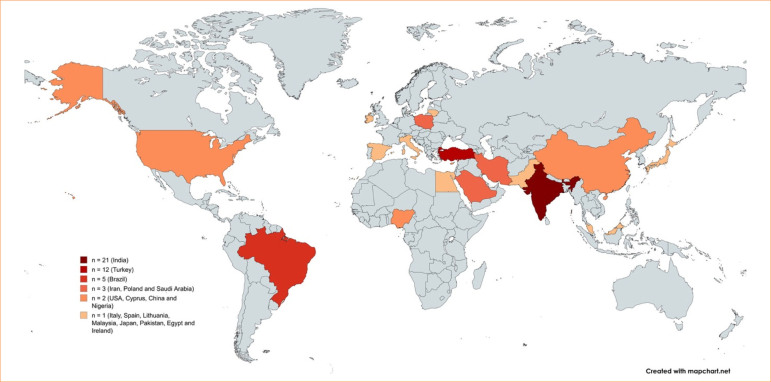
Number of publications on the topic of each country.

Source: Elaborated by the authors.

The publication range of the included articles spanned from 2009 to 2024. The geographical distribution revealed 21 studies conducted in India, 12 in Turkey, five in Brazil, three in Saudi Arabia, three in Poland, three in Iran, two in the United States of America, two in Cyprus, two in China, one in Italy, one in Spain, one in Malaysia, one in Lithuania, two in Nigeria, one in Japan, one in Pakistan, one in Egypt, and one in Ireland. Figure 2 shows the countries that most published on the topic.

### Blood concentrates and their impact on postoperative outcomes: an analysis of eligible studies

Most of the 63 eligible studies applied L-PRF, followed by PRP, A-PRF, CGF, platelet-rich growth factor (PRGF), injectable platelet-rich fibrin (i-PRF), advanced platelet-rich fibrin plus (A-PRF+), lyophilized platelet-rich plasma (LPRP), and albumin with platelet-rich fibrin (Alb-PRF).

The outcomes showed that most eligible studies assessed postoperative pain, followed by edema, trismus, alveolar osteitis, and infection. All analyzed outcomes presented contradictory findings when comparing the group performing only clot promotion with the one receiving the blood concentrate or groups comparing two blood concentrates. Table 3 presents the main results of the eligible studies.

**Table 3:** Main findings of the eligible studies.

Authors, year of publication, country	Groups	Outcomes				
		Pain	Edema	Trismus	Alveolar osteitis	Infection
Gawande and Halli, 2009^24^ India	Blood clot PRP	There was no statistically significant difference between the groups at 2 and 7 postoperative days	At 2 and 7 postoperative days, a statistically significant difference was observed in the mean percentage reduction of edema in the test group	n. r.	n. r.	n. r.
Vivek and Sripathi Rao, 2009^25^ India	Blood clot PRP	There was no statistically significant difference between the groups on days 1, 2, and 3	n. r.	n. r.	n. r.	n. r.
Arenaz-Búa et al., 2010^26^ Spain	Blood clot PRP	There was no statistically significant difference between the groups over the seven postoperative days	There was no statistically significant difference between the groups over the seven postoperative days	There was no statistically significant difference between the groups over the seven postoperative days	n. r.	The difference between the groups was not statistically significant
Mozzati et al., 2010^27^ Italy	Blood clot PRGF	Although the sites receiving PRGF showed lower pain scores, the difference was statistically significant only on the seventh postoperative day	The side subjected to PRGF treatment exhibited a statistically significant edema reduction on the second postoperative day	n. r.	n. r.	n. r.
Rutkowski et al., 2010^28^ United States of America	Blood clot PRP	There was no statistically significant difference between the groups	There was no statistically significant difference between the groups	n. r.	n. r.	n. r.
Ogundipe et al., 2011^29^ Nigeria	Blood clot PRP	There was a statistically significant difference for the test group during the 14 postoperative days (*p* < 0.001)	There was no statistically significant difference between the groups on postoperative days 1, 7, and 14	Although mouth opening was higher in the test group over the 14 postoperative days, there was no statistically significant difference	n. r.	n. r.
Singh et al., 2012^30^ India	Blood clot L-PRF	Although pain scores were lower in the test group on postoperative days 1, 2, and 3, there was no statistically significant difference between the groups	n. r.	n. r.	n. r.	n. r.
Hoaglin and Lines, 2013^31^ United States of America	Blood clot L-PRF	n. r.	n. r.	n. r.	The occurrence of alveolar osteitis was lower with statistical significance in patients receiving PRF, with p = 0.0001	n. r.
Eshghpour et al., 2014^32^ Iran	Blood clot L-PRF	n. r.	n. r.	n. r.	The frequency of alveolar osteitis in the PRF group was significantly lower (*odds ratio* = 0.44; *p* < 0.05).	n. r.
Gawai and Sobhana, 2015^33^ India	Blood clot PRP	There was a significant difference in pain on the sixth and seventh postoperative days in the test group	There was a significant difference in swelling on the sixth and seventh postoperative days in the test group	n. r.	n. r.	n. r.
Kumar et al., 2015^34^ India	Blood clot L-PRF	There was a statistically significant difference in pain on the first postoperative day in the test group (*p* = 0.017)	There was a statistically significant difference in edema on the first postoperative day in the test group (*p* = 0.022)	There was a statistically significant difference in trismus on the first postoperative day in the test group (*p* = 0.04)	n. r.	n. r.
Ozgul et al., 2015^35^ Turkey	Blood clot L-PRF	There were no statistically significant differences between the groups during the 7th postoperative day	There was lower edema with statistically significant measures on the first and third postoperative days on the side receiving PRF (*p* < 0.05)	n. r.	n. r.	n. r.
Uyanik et al., 2015^36^ Cyprus	Blood clot L-PRF + conventional surgery L-PRF + piezosurgery	There was a statistically significant difference over the 7 postoperative days for PRF + conventional surgery (*p* = 0.001) and PRF + piezosurgery (*p* = 0.0001) compared to the blood clot	There was a statistically significant difference only on the second postoperative day for PRF + conventional surgery (*p* = 0.018) and PRF + piezosurgery (*p*= 0.006) compared to the blood clot	There was a statistically significant difference only at 24 postoperative hours for PRF + conventional surgery (*p* = 0.011) and PRF + piezosurgery (*p* = 0.019) compared to the blood clot	n. r.	n. r.
Bilginaylar and Uyanik, 2016^37^ Cyprus	Blood clot (conventional surgery) L-PRF + conventional surgery Blood clot (piezosurgery) L-PRF + piezosurgery	There was a statistically significant difference over the 7 postoperative days for PRF + conventional surgery (*p* = 0.001) and PRF + piezosurgery (*p* = 0.0001)	There were no statistically significant differences between the groups	There were no statistically significant differences between the groups	n. r.	n. r.
Dutta et al., 2016^38^ India	Blood clot PRP L-PRF	During the 7 postoperative days, the maximum pain reduction occurred in patients receiving PRP and PRF	During the 7 postoperative days, the maximum edema reduction occurred in patients receiving PRP and PRF	n. r.	Alveolar osteitis occurred only on the third postoperative day in two patients from the control group, two patients treated with PRP, and one patient treated with PRF	n. r.
Kumar et al., 2016^39^ India	Blood clot L-PRF	There was a statistically significant difference in pain reduction in patients treated with PRF on the first, third, and seventh postoperative days	n. r.	n. r.	n. r.	Postoperative infection occurred in only one patient in the control group
Al-Hamed et al., 2017^40^ Egypt	Blood clot L-PRF	Patients receiving PRF showed a statistically significant pain reduction on the fifth (*p* = 0.041), sixth (*p* = 0.032), and seventh (*p* = 0.005) postoperative days	n. r.	n. r.	n. r.	n. r.
Asutay et al., 2017^41^ Turkey	Blood clot L-PRF	There were no statistically significant differences between the groups	There were no statistically significant differences between the groups	There were no statistically significant differences between the groups	Alveolar osteitis occurred in three patients on the control side and in only one patient on the test side	n. r.
Gandevivala et al., 2017^42^ India	Blood clot PRP	There was a statistically significant pain reduction on the third and seventh postoperative days on the test side	There was a statistically significant edema reduction on the third postoperative day on the test side	n. r.	n. r.	n. r.
Gülşen and Şentürk, 2017^43^ Turke	Blood clot L-PRF	There were no statistically significant differences between the groups	There were no statistically significant differences between the groups	n. r.	n. r.	n. r.
Rastogi et al., 2018^44^ India	L-PRF	There was a significant pain reduction for all patients on the third and seventh postoperative days (*p* < 0.05)	n. r.	n. r.	n. r.	n. r.
Afat et al., 2018^45^ Turkey	Blood clot L-PRF	There were no statistically significant differences between the groups	L-PRF, particularly when combined with hydroxyapatite, seems an effective alternative for edema control	There were no statistically significant differences between the groups.	n. r.	n. r.
Bhujbal et al., 2018^46^ Iran	Blood clot PRP	There was no statistically significant difference between the groups immediately after surgery or on the first, third, and seventh postoperative days	The experimental side showed a statistically significant reduction on the first (*p* = 0.0059) and third (*p* = 0.0001) postoperative days	n. r.	n. r.	n. r.
Dar et al., 2018^47^ India	Blood clot L-PRF	The test side showed a statistically significant pain reduction throughout the evaluated period (*p* < 0.05)	The test side showed a statistically significant edema reduction throughout the evaluated period (*p* < 0.001)	n. r.	n. r.	n. r.
Daugela et al., 2018^16^ Lithuania	Blood clot L-PRF	During the first week of postoperative recovery, there was a significant pain reduction on the test side (*p* = 0.001)	The test side showed a significant edema reduction on the first (*p* = 0.035) and third (*p* = 0.023) postoperative days	n. r.	n. r.	n. r.
Eshghpour et al., 2018^48^ Iran	Blood clot L-PRF	n. r.	n. r.	n. r.	The test side showed a statistically lower frequency of alveolar osteitis than the control side	n. r.
Jeyaraj and Chakranarayan, 2018^49^ India	Blood clot L-PRF	The group receiving L-PRF reduced pain	The group receiving L-PRF exhibited reduced edema	The group receiving L-PRF showed reduced trismus	n. r.	n. r.
Afat et al., 2019^50^ Turkey	Blood clot L-PRF	n. r.	n. r.	n. r.	Only one patient from the control group presented postoperative alveolar osteitis. There were no cases of alveolar osteitis in the test group	Only one patient from the control group presented postoperative infection. There were no cases of infection in the test group
Caymaz and Uyanik, 2019^51^ Turkey	L-PRF A-PRF	Pain scores were significantly higher in the L-PRF group than in the A-PRF group on the first, second, and third postoperative days (*p* < 0.05). There were no differences between the groups on the seventh day	There were no statistically significant differences between the groups during the evaluated period	There were no statistically significant differences between the groups during the evaluated period	n. r.	n. r.
Kapse et al., 2019^52^ India	Blood clot L-PRF	Pain significantly reduced on the test side throughout the evaluated period (*p* < 0.05)	Edema significantly reduced on the test side throughout the evaluated period (*p* < 0.05)	n. r.	n. r.	n. r.
Ritto et al., 2019^53^ Brazil	Blood clot L-PRF	There were no statistically significant differences in pain reduction between the groups	n. r.	n. r.	n. r.	n. r.
Zahid and Nadershah, 2019^54^ Saudi Arabia	Blood clot A-PRF	The side treated with A-PRF showed a statistically significant pain reduction on the seventh postoperative day	The side treated with A-PRF showed a statistically significant edema reduction on the seventh postoperative day	n. r.	n. r.	n. r.
Aftab et al., 2020^55^ India	Blood clot PRP	The first, third, fifth, and seventh postoperative days showed a significant pain reduction in patients receiving PRP (*p* < 0.05)	The first, third, fifth, and seventh postoperative days showed a significant edema reduction in patients receiving PRP (*p* < 0.05)	There was a noticeable increase in interincisal opening (*p* = 0.001) in the experimental group compared to the control group	n. r.	
Bhujbal et al., 2020^56^ India	PRP L-PRF	There was no significant difference in pain reduction in the immediate postoperative period (*p* < 0.15) and on the first (*p* < 0.96), third (*p* < 0.58), and seventh (*p* < 0.78) days	There was a significant edema reduction on the first (*p* < 0.0020) and third (*p* < 0.0010) days at the L-PRF site, but it ceased without significant difference on the seventh day (*p* < 1.00)	n. r.	n. r.	n. r.
Özveri Koyuncu et al., 2020^57^ Turkey	Blood clot CGF	n. r.	n. r.	n. r.	The incidence of alveolar osteitis in the control group (n = 8; 11.4%) was higher than in the test group (n = 0), and the difference between study groups was statistically significant on the third postoperative day (*p* < 0.001).	n. r.
Özveri Koyuncu et al., 2020^58^ Turkey	Blood clot CGF	Postoperative intensity in the CGF group was lower than in the control group for seven days (*p* < 0.001)	Total edema in the CGF group was lower than in the control group, and there was a significant difference between them in temporal interactions (*p* < 0.001)	The mean mouth-opening values significantly differed between the groups at three and seven days, with better outcomes for the CGF group (*p* < 0.001)	n. r.	n. r.
Kumar et al., 2020^59^ India	Blood clot PRP	The pain score on the control side was higher than on the side receiving PRP	Tragus-subnasal and tragus-pogonion edema was higher on the control side than on the side receiving PRP	The mean trismus on the side receiving PRP was significantly lower (p < 0.001) than the control side	n. r.	n. r.
Miyamoto et al., 2020^60^ Japan	Blood clot L-PRF	On the third and the fourth postoperative days, the side receiving L-PRF presented significant pain reduction compared to the control side	Although the side receiving L-PRF showed lower edema, there was no statistically significant difference compared to the control side	n. r.	n. r.	n. r.
Sybil et al., 2020^61^ India	Blood clot L-PRF	There were statistically significant differences in pain intensity on the control sides and tests at the first, third, and seventh days (*p* < 0.001)	Edema was constantly lower with statistical significance (*p* < 0.001) on the test side than on the control side	n. r.	n. r.	n. r.
Torul et al., 2020^62^ Turkey	Blood clot A-PRF CGF	There were no statistically significant differences in pain among the groups	The change in tragus-to-labial commissure measurements was significantly different at baseline-7th day between control and CGF groups (*p* = 0.038). There were significant differences between tragus-to-pogonion measurements at baseline-7th day between control-CGF groups (*p* = 0.014) and A-PRF-CGF groups (*p* = 0.038)	There was no significant difference among groups	n. r.	n. r.
Gupta and Agarwal, 2021^63^ India	Blood clot A-PRF	The data from day 1 does not show statistically significant differences between test and control groups, but values from days 3 and 7 were statistically significant, favoring the A-PRF group	At day 1, the mean edema scores were 2.43 and 2.61, with *p* = 0.061, indicating no statistical significance. However, the scores at day 3 (3.46 and 3.71) were significant	At day 1, the values were not statistically significant. The values at day 3 were highly significant with *p* = 0.0001, indicating a noticeable mouth-opening improvement when using A-PRF	n. r.	n. r.
Hanif and Sheikh, 2021^64^ Pakistan	Blood clot PRP	Pain was lower in the PRP group than in the non-PRP group according to the visual analog scale, on the seventh follow-up visit (*p* < 0.05)	n. r.	Trismus was lower in the PRP group than in the non-PRP group (*p* < 0.05)	n. r.	n. r.
Nourwali, 2021^65^ Saudi Arabia	Blood clot L-PRF	There was no significant difference among groups	There was no significant difference among groups	n. r.	n. r.	n. r.
da Silva et al., 2021^66^ Brazil	Blood clot L-PRF	The mean postoperative pain score was lower for the L-PRF group at all times, showing statistical significance (*p* < 0.05)	n. r.	n. r.	n. r.	n. r.
Nowak et al., 2021^67^ Poland	Blood clot L-PRF	Pain perception significantly differed on the seventh postoperative day in the study group compared to the control group	The difference in edema occurrence between the groups was statistically significant. The mean frequency of edema in the study group was 0.1 ± 0.31, whereas in the control group it was 0.53 ± 0.51	Trismus significantly differed on the seventh postoperative day in the study group compared to the control group	n. r.	n. r.
Starzyńska et al., 2021^68^ Poland	Blood clot A-PRF	Pain was significantly less intense in the study group on the third (*p* < 0.001) and seventh (*p* < 0.001) postoperative days	There was a statistically significant difference favoring the study group on the third (*p* < 0.001) and seventh (*p* < 0.001) postoperative days	There was a statistically significant difference favoring the study group on the third (*p* < 0.001) and seventh (*p* = 0.001) postoperative days	There was no association between A-PRF application and alveolitis prevalence	n. r.
Trybek et al., 2021^69^ Poland	Blood clot L-PRF	The pain level significantly differed (*p* < 0.05) six hours and on days 1 and 3 postoperatively between patients in the study and control groups. A notable disparity in pain sensation occurred on the first postoperative day, with participants in the control group experiencing higher pain intensity	There was no statistically significant difference in edema reduction between the groups during the evaluated period	The study and control groups significantly differed (p < 0.05) in mouth opening after days 1, 2, and 7. Mouth opening was significantly higher on postoperative days 1, 2, and 7 in the study group compared to the control group	n. r.	n. r.
Elayah et al., 2022^70^ China	Blood clot CGF	Although there was no statistically significant difference in pain scores between sides on the first postoperative day, the test side showed a significant reduction in pain scores on the third and seventh days compared to the control (*p* = 0.001 and *p* < 0.001, respectively)	The test side showed significantly lower edema than the control side, especially on the first (1.01 ± 0.57 vs. 1.55 ± 0.56) and third (1.42 ± 0.8 vs. 2.63 ± 1.2) postoperative days. However, edema disappeared by the seventh postoperative day on both sides	n. r.	n. r.	n. r.
Fang et al., 2022^71^ China	Blood clot CGF	The postoperative pain score at 2, 24, and 48 hours showed a significantly lower pain score in the CGF group than in the control group	There was no significant difference in edema reduction between the two groups at 48 hours or one week	There was no significant difference in trismus reduction between the two groups at 48 hours or one week	None of the patients experienced dry socket in the CGF group. Three patients suffered from dry socket in the control group	n. r.
Konuk and Senturk, 2022^72^	Blood clot L-PRF	n. r.	Although the level of edema was lower in the PRF group than in the non-PRF group on all days, the results were not statistically significant (*p* > 0.05)	n. r.	n. r.	n. r.
Osagie et al., 2022^73^ Nigeria	PRP L-PRF	There was a statistically significant pain reduction in the group receiving L-PRF compared to the group receiving PRP	There was no statistically significant difference in edema reduction between the groups during the evaluated period	There was no statistically significant difference in trismus reduction between the groups during the evaluated period	n. r.	n. r.
O’Sullivan et al., 2022^74^ Ireland	Blood clot PRGF	The mean pain score on the third day was higher in the PRGF group (4.1 ± 2.4) than in the control group (3.2 ± 2.3), demonstrating borderline significance (mean difference = 1.0; *p* = 0.06). There was no significant difference in the mean pain score on the seventh day between the PRGF (2.7 ± 2.2) and control (3.2 ± 2.6) groups (*p* = 0.44)	n. r.	Reduced mouth opening occurred on the seventh day in the control (35.7 ± 8.2 mm) and PRGF (35.4 ± 8.5 mm) groups without differences between the groups (*p* = 0.67)	Four patients developed alveolar osteitis postoperatively, one in the control group (3%) and three in the PRGF group (9%)	n. r.
Rengarajoo et al., 2022^75^ Malaysia	Blood clot LPRP	There was no significant difference in reported pain scores between the two groups. However, the L-PRP-treated group recorded a slightly higher pain score over the first seven days	There was no significant difference in facial edema size between the L-PRP-treated group and the control group. However, the L-PRP-treated group recorded slightly higher edema throughout the study period	Interincisal mouth opening significantly reduced on the first (17.13 mm) and second (19.8 mm) postoperative days. Mouth opening significantly increased to 27.2 mm on the seventh day compared to the first day (*p* < 0.001)	n. r.	n. r.
Riaz et al., 2022^76^ India	L-PRF A-PRF	The A-PRF group showed a significantly higher pain reduction than the L-PRF group (*p* = 0.063)	The A-PRF group exhibited a significantly higher edema reduction than the L-PRF group (*p* = 0.001)	The A-PRF group showed a significantly higher trismus reduction than the L-PRF group (*p* = 0.013)	n. r.	n. r.
Sargaiyan et al., 2022^77^ India	Blood clot PRP	PRP exhibited a significant pain reduction on the first (*p* = 0.002) and second (*p* = 0.091) postoperative days	There was no statistically significant difference in edema reduction between the groups	n. r.	Alveolar osteitis did not occur in either group during the evaluated period	n. r.
Shruthi et al., 2022^78^ India	Blood clot L-PRF	The mean postoperative pain intensity was 88.665 in the study group and 55.578 in the control group. Pain scores were 4.91 ± 1.51 on the first postoperative day and 1.73 ± 0.63 on the seventh postoperative day in the control group, whereas in the study group they were 4.86 ± 1.78 and 0.68 ± 0.57, respectively	The mean postoperative edema in the control group was 94.282 mm, and 141.099 mm in the study group. Edema measured 352.36 ± 23.2 mm in the control group and 349.5 ± 23.1 mm in the study group on the first postoperative day. These measurements were 353.18 ± 25.7 mm in the control group and 348.8 ± 26.1 mm in the study group on the seventh postoperative day	The mean preoperative mouth opening was 34.351 mm in the study group and 52.328 mm in the control group. Mouth opening measured 39.23 ± 6.57 mm in the control group on the first postoperative day and 41.45 ± 6.32 mm on the seventh postoperative day. In the study group, the corresponding measurements were 42.00 ± 5.33 mm and 44.23 ± 5.07 mm	n. r.	n. r.
Asif et al., 2023^79^ Saudi Arabia	Blood clot L-PRF	There were no statistically significant differences between the groups	n. r.	n. r.	Group I (PRF group) showed a dry socket prevalence after lower third molar surgery of 2.22 (two patients), and Group II (non-PRF group) had 12.22 (nine patients), both with *p* = 0.010	n. r.
Javid et al., 2023^80^ Brazil	L-PRF Alb-PRF	There was no significant difference between L-PRF and Alb-PRF groups during the same experimental period (*p* < 0.05)	There was no statistical difference between L-PRF and Alb-PRF groups during the evaluated periods (*p* > 0.05)	Mouth opening limitation significantly reduced seven days after surgery (2.32; 95% confidence interval 1.96-2.68) compared to the first post-surgery day (2.77 mm; 95% confidence interval 2.14-3.39) (*p* = 0.03)	n. r.	n. r.
Karaca et al., 2023^81^ Turkey	Blood clot L-PRF	Pain was significantly lower on the second and the seventh postoperative days in the PRF group than in the control group (*p* < 0.001, *p* = 0.002, respectively)	Edema levels were significantly lower in the PRF group in the LC-G measurements on the second and the seventh postoperative days (*p* < 0.001, p = 0.026, respectively). The T-AC measurements on the second postoperative day also showed significantly lower results in the PRF group (*p* = 0.021)	The trismus evaluation showed a significantly higher interincisal distance in the PRF group on the second and the seventh postoperative days compared to the control group (*p* < 0.001, *p* < 0.001, respectively).	n. r.	n. r.
Pereira et al., 2023^82^ Brazil	Blood clot A-PRF+	The clinical analysis revealed a gradual pain reduction in both groups at 90 days compared to the seventh postoperative day. However, there were no differences between the extraction sockets treated or not with A-PRF+	The clinical analysis revealed a gradual edema reduction in both groups at 90 days compared to the seventh postoperative day. However, there were no differences between the extraction sockets treated or not with A-PRF+	n. r.	n. r.	n. r.
Rodrigues et al., 2023^83^ Brazil	Blood clot L-PRF	Pain scores showed statistically significant differences between PRF and control groups at 6 (*p* = 0.001), 8 (*p* = 0.001), 16 (*p* = 0.025), 24 (*p* < 0.001), 48 (*p* < 0.001), and 72 (*p*< 0.001) hours, with the control side showing higher pain scores	Regardless the group, edema peaked 72 hours after surgery. The PRF sides showed statistically significant lower means at this postoperative time point (*p* < 0.001)	n. r.	n. r.	n. r.
Kalyani et al., 2023^84^ India	Blood clot L-PRF	Postoperative pain on the first, third and seventh postoperative days revealed a significant difference between L-PRF and control sides on the first and seventh postoperative days, with a lower mean pain score on the L-PRF side	Postoperative edema on the first, third and seventh postoperative days revealed a significant difference between L-PRF and control sides on the first and third postoperative days, with a reduction in the mean swelling on the L-PRF side	n. r.	n. r.	n. r.
Akpinar and Ayrancı, 2024^85^ Turkey	Blood clot i-PRF	There was no statistically significant difference at any time point (p > 0.05)	Significantly lower differences occurred on the second and seventh postoperative days in the measurements from the outer corner of the eye to the mandible angle on the side treated with i-PRF. Additionally, measurements from the tragus to the outer corner of the mouth on the i-PRF side were smaller on the second day, with statistical significance	Maximum mouth opening was significantly lower on the i-PRF side on the second postoperative day (control 27.88 ± 6.48, i-PRF 25.51 ± 5.56) (*p* = 0.025). However, there was no statistically significant difference between the sides on the seventh day (*p* > 0.05)	n. r.	n. r.

n. r.: not reported; PRP: platelet-rich plasma; L-PRF: leukocyte- and platelet-rich fibrin; A-PRF: advanced platelet-rich fibrin; A-PRF+: advanced platelet-rich fibrin plus; CGF: concentrated growth factor; Alb-PRF: albumin with platelet-rich fibrin; i-PRF: injectable platelet-rich fibrin; PRGF: platelet-rich growth factor; PRF: platelet-rich fibrin.

**Figure 3:**
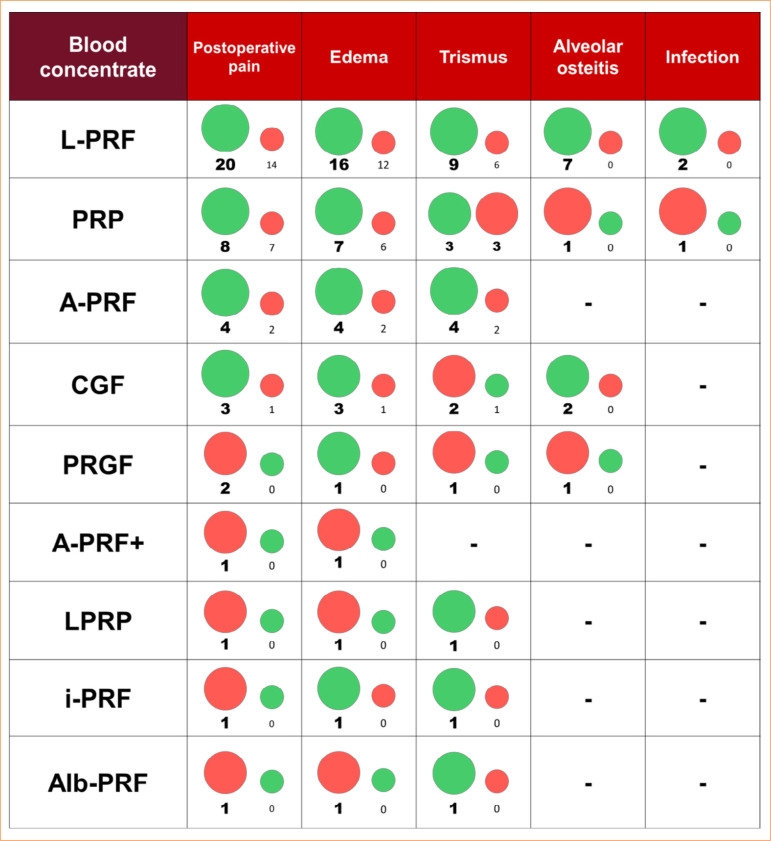
Summary diagram of the performance of blood concentrates according to statistical analysis. Green circles indicate studies in which there were statistically significant differences in the improvement of inflammatory signs and symptoms after third molar extraction, and red circles indicate studies in which there was no statistically significant difference. Larger circles represent the prevalence of results. L-PRF: leukocyte- platelet-rich plasma; PRP: platelet-rich plasma; A-PRF: advanced platelet-rich fibrin; CGF: concentrated growth factor; PRGF: plasma rich in growth factos; A-PRF+: advanced platelet-rich fibrin plus; LPRP: lyophilized platelet-rich plasma; i-PRF: injectable platelet-rich fibrin; Alb-PRF: albumin with platelet-rich fibrin.

Source: Elaborated by the authors.

Figure 3 presents a summary diagram of the results of the performance of blood concentrates according to the statistical analysis.

## Discussion

The present scoping review evaluated the current knowledge of blood concentrates for managing inflammatory signs and symptoms and the complications from third molar extractions. It also analyzed the countries with the most scientific production in this field and the primary researchers and journals that most frequently published articles.

Considering the complexity of this procedure, third molar removal may cause a postoperative period characterized by intense pain, edema, mouth opening difficulty, alveolar osteitis, and infection^5^,^50^,^81^. Alveolar healing involves a complex and highly coordinated sequence of cell and molecular responses to restore tissue integrity. Although the literature findings are contradictory, blood concentrates have been extensively applied to improve this step and optimize the postoperative inflammatory process^86^,^87^.

Promising outcomes from applying blood-derived products in the surgical context, such as fibrin adhesives, encouraged studies promoting the development of PRP, the first explored blood concentrate^88^,^89^. This blood product contains more than 95% of platelets, high-platelet growth factor, coagulation factor, and cytokine levels. These components demonstrate the capacity to optimize tissue repair by stimulating angiogenesis and cell proliferation and activating cells related to wound healing, such as fibroblasts, neutrophils, and mesenchymal stem cells^90^,^91^.

Although PRP appears in several of the included studies, the findings were inconsistent regarding its performance in controlling the inflammatory process and postoperative complications^46^,^64^,^77^. Ogundipe et al.^29^ showed that PRP application to sockets after lower third molar extraction promoted a lower mean score of postoperative pain, and there were no statistically significant differences in edema and trismus reduction compared to the control group. These findings contradict Gandevivala et al.^42^, whose topical PRP application caused a statistically significant edema reduction, and there were no statistically significant differences in pain between the test and control sides.

Considering some limitations in obtaining PRP, such as prolonged clinical time, the need for adding procoagulant, and especially the fast and unsupported release of components that optimize tissue repair, studies have searched for new blood-derived products^92^. Thus, in 1999, Anitua^93^ developed PRGF. The PRGF handles several limitations inherent to PRP preparation, such as bovine thrombin replacement with calcium or autologous thrombin as an activator, preventing immune reactions and the risk of disease transmission^94^,^95^. Mozzati et al.^27^ determined that PRGF was statistically effective in controlling pain only on the seventh postoperative day, and edema significantly reduced only on the second day. However, O’Sullivan et al.^74^ did not find statistically significant differences in pain, trismus, and alveolar osteitis reduction compared to the control group during the analysis period. PRGF differs from PRP in the selective leukocyte exclusion during preparation, aiming at a homogeneous platelet distribution^96^. Yet, these two blood concentrates did not show superiority regarding physical properties, physiological performance, or clinical outcomes^74^.

The second-generation concentrate known as L-PRF represented a breakthrough for new blood concentrate development^12^. However, the literature also presents contradictory findings about the role of L-PRF in controlling inflammatory signs and symptoms. Kalyani et al.^84^ demonstrated that L-PRF was effective with statistical significance in reducing pain in the first and seventh postoperative days. Daugela et al.^16^ attributed that to platelet growth factors, such as the platelet-derived growth factor, insulin-like growth factor, vascular endothelial growth factor, and the transforming growth factor-beta. Although Asutay et al.^41^ affirmed that this blood concentrate benefits tissue repair, the findings of this study did not show statistically significant differences in pain, edema, and trismus compared to the control group after lower third molar extraction. These authors potentially associated divergent findings with sample heterogeneity, different flap designs, and surgical difficulty levels^41^. Bolukbasi et al.^97^ and Pathak et al.^98^ affirmed that L-PRF presents high bioabsorption and lower stability, potentially harming growth factor release in the long term. Thus, aiming to reduce bioabsorption and increase L-PRF stability, a new concentrate was developed by adding the liquid portion of L-PRF to the denatured platelet-poor plasma, generating Alb-PRF^99^. However, Javid et al.^80^ did not find statistically significant differences in pain, edema, and trismus reduction compared to L-PRF.

Wend et al.^100^ showed inconsistency in centrifugation forces to obtain L-PRF, potentially affecting its composition and efficacy. The programmed alternation between acceleration and deceleration is superior to fixed centrifugation forces because it increases platelet collision with the centrifugation tubes, promoting a fibrin network with higher tensile strength and a prolonged release of platelet growth factors^101^,^102^. This concept allowed the development of a CGF, also known as L-PRF-derived or Sacco’s PRF^103^. Except for Fang et al.^71^, who did not find statistically significant differences in edema and trismus reduction, CGF significantly reduced pain, edema, trismus, and alveolar osteitis incidence in the other studies included in the present review^70^,^104^,^105^. Although CGF efficacy seems superior to L-PRF, these outcomes must be carefully interpreted. The centrifugation protocol and factors such as sample pattern and surgical complexity may also influence the clinical response of blood concentrates^82^,^105^.

The concept of low-speed centrifugation emerged to increasingly improve tissue repair, promoting the development of blood concentrates, such as i-PRF, A-PRF, and A-PRF+^12^,^104^. Only one study in this review analyzed i-PRF performance, indicating that this blood concentrate did not significantly reduce pain and trismus during the analysis period^85^. Besides the statistically significant pain reduction on the third postoperative day, Gupta and Agarwal^63^ also showed that A-PRF promoted statistically significant edema and trismus reduction after lower third molar extraction relative to the control group. *In-vitro* studies demonstrated that this new generation of blood concentrates has a more porous three-dimensional fibrin matrix, a homogeneous and more concentrated cell distribution, and the possibility of releasing growth factors, potentially making this matrix superior to L-PRF in some clinical conditions^99^,^106^. Pereira et al.^82^ analyzed the influence of A-PRF+ on pain and edema reduction after upper third molar extraction. They found no statistically significant differences in both variables compared to the control group. These authors potentially attribute the outcomes to favorable clinical conditions, such as upper third molar positioning relative to the second molar’s occlusal level and the low complexity of the surgical procedure^82^.

Taken together, the results demonstrate the growing interest in improving surgical outcomes through blood concentrates. However, despite these advances in the application of blood concentrates in tissue repair, the methodological heterogeneity among studies limits the interpretation of results. Standardizing criteria such as participant age, sample size, third molar positioning, study design, surgical complexity, and blood concentrate preparation protocols-including tube type, relative centrifugation force, and time-is essential to enable more robust comparisons and generate reliable evidence.

## Conclusion

Most included studies did not standardize third molar positioning or centrifugation protocols to obtain blood concentrates. Moreover, the studies present different designs, some comprising parallel groups and others employing split-mouth. The comparison of inflammatory signs and symptoms among different individuals might introduce an outcome interpretation bias compared to the split-mouth analysis. However, several split-mouth studies did not apply a weekly interval between the operated sides, potentially introducing an outcome interpretation bias.

The literature shows that blood concentrates present biological properties that favor tissue repair. However, the published studies have conflicting findings regarding the performance of these products in controlling inflammatory signs and symptoms. Considering the heterogeneity of the articles, a methodological standardization of randomized clinical trials is recommended to obtain robust outcomes on the topic.

## Data Availability

All data sets were generated or analyzed in the current study.
